# Folic acid supplementation in pregnancy and implications in health and disease

**DOI:** 10.1186/s12929-014-0077-z

**Published:** 2014-08-19

**Authors:** Subit Barua, Salomon Kuizon, Mohammed A Junaid

**Affiliations:** 1 Department of Developmental Biochemistry, New York State Institute for Basic Research in Developmental Disabilities, 1050 Forest Hill Road, Staten Island 10314, NY, USA

**Keywords:** Folic acid, DNA methylation, Epigenetic, Imprinting, Prenatal nutrition, Neural tube defects, Autism

## Abstract

Maternal exposure to dietary factors during pregnancy can influence embryonic development and may modulate the phenotype of offspring through epigenetic programming. Folate is critical for nucleotide synthesis, and preconceptional intake of dietary folic acid (FA) is credited with reduced incidences of neural tube defects in infants. While fortification of grains with FA resulted in a positive public-health outcome, concern has been raised for the need for further investigation of unintended consequences and potential health hazards arising from excessive FA intakes, especially following reports that FA may exert epigenetic effects. The objective of this article is to discuss the role of FA in human health and to review the benefits, concerns and epigenetic effects of maternal FA on the basis of recent findings that are important to design future studies.

## Review

### Introduction

The emerging view of epidemiological studies indicates the importance of the intrauterine environment in early fetal development. With increased understanding of the fundamental mechanism, appropriate DNA methylation including the proper functioning of the epigenetic machinery is highlighted to be essential for embryogenesis and adult health [1]-[3]. Thus, FA has gained considerable attention because of its promising role in modulating diverse clinical conditions, whereas folate deficiency has been linked with a variety of disorders including birth defects and defects in the development of neural tube closure [4]-[6]. As stated by Hippocrates nearly 2,500 years ago: *“Let food be thy medicine and medicine be thy food”* the mandatory FA fortification appears to be a first modern attempt to design a strategy for using food for the prevention or treatment of developmental defects. However, this mandate has resulted in an increase in folate level in the serum to approximately 19 ng/ml, which is above the normal range of the serum folate concentrations in humans i.e. 2.7-17 ng/ml [7]. Epidemiological studies have shown that a significant number of women who took FA supplements during pregnancy exceeded the Institute of Medicine's recommended tolerable upper limit of 1,000 μg/day. In addition, studies also reported consumption of 400 μg/day of natural food folate plus FA-containing prenatal supplements resulted in supra-nutritional folate status with the greatest increases in pregnant women followed by lactating and non-pregnant women [8],[9]. Concern has been raised if such exposure as a result of FA fortification will have any detrimental effects in the general population if not overtly benefit. This review summarizes the beneficial role of folate in human health, the metabolic pathway, epigenetic mechanism and potential concerns based on recent findings.

### Folic acid and neural tube defects

Birth defects are one of the major burdens in the human public health with estimates from Centers for Disease Control and Prevention (CDC) approaching 1 in every 33 newborns in the US and accounting for more than 20% of all infant mortalities [10],[11]. Neural tube defects (NTDs) are common complex multifactorial disorders in the neurulation of the brain and spinal cord that occurs between 21 and 28 days after conception in humans [12]. Worldwide depending on the ethnic grouping and geographical location, the prevalence has been reported to vary widely between 1and 10 in every 1000 births or established pregnancies [13]. While we are beginning to understand the underlying etiologies, evidence gathered so far implicates both -genetic and non-genetic factors such as maternal nutritional status or maternal obesity in the onset of NTDs [14]-[16]. Over the years, numerous studies including community-based trials often suggested NTDs as vitamin deficiency disorders and have shown that the exogenous or periconceptional supplementation of maternal FA can reduce the risk of NTDs in offspring [17]. Indeed, research spanning decades suggests folate deficiency as a risk factor of NTDs; however the involvement of whole methylation metabolism has also been linked with the etiology of NTDs [16],[18],[19]. Arguing against the maternal folate deficiency model alone, some studies also reported normal concentrations of folate in the mothers of human fetuses with NTDs. Supporting this, studies in cultured rat embryos or FA deficient mice were reported not to be affected by NTDs as a result of FA deficiency [20]-[24]. In contrast, studies also reported that exogenous FA and thymidine in the homozygous *splotch* (*Pax3*) mouse embryos prevented NTDs and corrected biosynthetic defects [25]. Therefore, no consensus has been reached based on the published data to date. However, as FA deficiency may be a risk factor for NTDs additional studies will be required to determine the mechanistic role of the FA pathway in the onset of neural tube defects.

### History and impact of folic acid on public health

A possible relationship between apparent folate deficiency and increased incidence of prematurity was suggested as early as 1944 by Callender [26]. This was later confirmed by Gatenby and Lillie [27], and in 1960s, Richard Smithells and Elizabeth Hibbard hypothesized that the under nutrition or impaired folate status could be an important factor in the origin of NTD based on significant observations, that women who had given birth to the children with birth defects i.e. anencephaly and spinabifida have an altered formiminoglutamic acid compared to the women with unaffected children [28]. To test this hypothesis Smithells and his group conducted an intervention trial with supplementation of a multivitamin containing diet with FA 0.36 mg/day during the periconceptional period to the participating women who previously had infants with NTD. In-contrast, women who were already pregnant without vitamin supplementation were considered as controls. In the1980’s, they published the results of this multi-center intervention study that revealed about 83-91% reduction in NTD recurrence in supplemented women compared to that of unsupplemented women [29]-[32]. These results first highlighted that multivitamin or FA supplementation may play a significant role in gestation and may reduce the recurrence of NTD. Later in 1991, after a randomized control trial (RCT) conducted at 33 centers in seven countries, the British Medical Research Council suggested, for women with a previous history of NTD-affected -pregnancies, the daily supplementation of 400 micrograms of FA is effective in preventing the recurrence of NTDs by 70% [33]. This was further supported by the results of a -RCT conducted in Hungary in 1992 that reported a daily intake of 0.8 mg of FA during the periconceptional period significantly reduced the incidence of a first occurrence of NTD [34]. In 1991, the CDC recommended a daily intake of 4000 μg of FA before and throughout the period of pregnancy for women with prior history of NTD-affected pregnancy [35]. Later in 1998, based on the evidence and recommendation from the wider medical community, the U.S. Public Health Service and Food and Drug Administration recommended mandatory fortifications of FA in flour and grains to prevent NTD and birth defects [36]. In 2007, the Canadian recommendations also included obesity (BMI >35) as a health risk, and recommended “the higher dose FA strategy (5 mg)” in patients with a history of poor compliance with medications and additional lifestyle issues of variable diet, no consistent birth control, alcohol, tobacco, and recreational non-prescription drugs use. Furthermore, to prevent the occurrence of NTDs in epileptic and diabetic mothers the recommendation is to take a higher dose of FA, 4–5 mg/day [37]-[39].

### Folate metabolism

**Figure 1:**
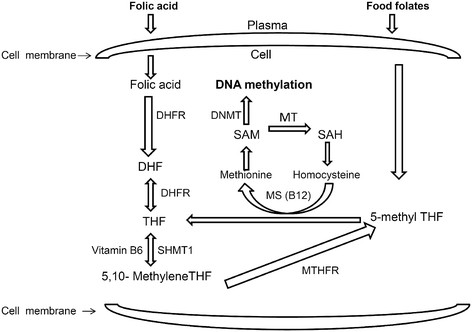
**Summary of folate metabolism (simplified).** The schematic diagram shows that after entry of synthetic FA or natural dietary folates through receptors/carriers in cell membrane, the intracellular folate/ FA pass through series of biochemical reactions, and alter DNA methylation. Abbreviations: DHFR, dihydrofolate reductase; DHF, dihydrofolate; THF, tetrahydrofolate; SHMT, serine-hydroxymethyltransferase; 5,10-methenyl- THF, 5,10-methenyl-tetrahydrofolate; MTHFR, 5,10-methylenetetrahydrofolate reductase; 5-methyl THF, 5-methyltetrahydrofolate; MS, methionine synthase; B12, vitamin B-12; SAM, *S*-adenosylmethionine; SAH, *S*-adenosylhomocysteine; DNMT, DNA methyltransferase; MT, methyltransferases.

FA is central to folate-requiring one-carbon metabolism which play key roles in numerous cellular reactions. These involve amino acid metabolism, biosynthesis of purine and pyrimidine; (the building blocks for DNA and RNA synthesis), and formation of primary methylating agent S-adenosyl-methionine (SAM), which is the universal methyl donor for DNA, histones, proteins and lipids [10]. Mechanistically, the transport of transmembrane folate is facilitated by both receptors and specific carriers active across cell membranes [40]. Under normal circumstances, natural dietary folate is absorbed in the intestine and/or liver and metabolized primarily to 5-methyl tetrahydrofolate (5-methylTHF) and subsequently gets polyglutamated for cellular retention (Figure 1). However, FA consumed in fortified foods/supplements is reduced primarly to dihydrofolate by the enzyme dihydrofolate reductase in the liver and finally converted to the tetrahydrofolate (THF), the substrate for polyglutamate synthetase. The polyglutamyl form of tetrahydrofolate (THF) formed either from FA or normal dietary folate is the central folate acceptor molecule in the one-carbon cycle. Next, THF is converted to 5,10-methyleneTHF by vitamin B6 dependent serine hydroxymethyltransferase and then reduced irreversibly to 5-methylTHF by methylenetetrahydrofolate reductase (MTHFR). 5-Methyl-THF acts as a primary methyl donor for the remethylation of homocysteine to methionine. Methonine is a key substrate for S-adenosylmethionine (SAM) which plays a central role in methylation reactions catalyzed by DNA methyltransferases (DNMTs) forming 5-methylcytosine [41]-[43]. Thus, the entire FA metabolism is modulated by several folate coenzymes. Mechanistically, the central role of this coenzyme is to modulate the metabolic pathway by accepting or donating one-carbon units [44]. Key genes in this pathway that are involved in transferring the methyl group to homocysteine, and have been most extensively studied include methylenetetrahydrofolate reductase (MTHFR), methionine synthase reductase (MTRR), reduced folate carrier (RFC), along with vitamin B12- dependent methionine synthase (MTR) [45]. Intriguingly, the etiology of NTDs has long been genetically associated with the dysregulation of the major folate pathway or methionine synthase genes, and single-nucleotide polymorphisms (SNPs) such as 677C > T in the MTHFR gene in humans [25],[46]. Thus, further studies on FA induced methylation and detailed analysis of the folate pathway and its role in mammalian neural tube closure could give us more insights in coming years.

### Implications of FA in epigenetic regulation

The possible impact of nutritional supplements, for example FA, on the mammalian genome can have long lasting effects in human health without any underlying genetic change. The availability of many dietary components involved in one-carbon metabolism including vitamin B6, choline, betanine, methionine, vitamin B12 and folate can result in alterations in the DNA methylation and histone modification. Mechanistically, the modulation of methylation patterns depends on the level of two metabolites of one-carbon metabolism: *S*-adenosylmethionine (SAM), a methyl donor and *S*-adenosylhomocysteine (SAH), a product inhibitor of methyltranferases [47]-[49]. Thus, nutrient epigenetic factors such as FA, a cofactor in one-carbon metabolism during gestation can affect the fetal programming and may modulate the genome-wide methylation pattern of DNA and cause dysregulation in the expression of genes [50]. The epigenetic impact of FA along with other one-carbon metabolites is best studied in the agouti mouse (*A*^*vy*^) experiment that has shown that the dietary methyl donors, including FA, has no affect on the *A*^*vy*^ methylation in the mother but clearly affected the *A*^*vy*^ methylation and phenotype of developing offspring [51]. Similar to the animal study, a study in young children from mothers having periconceptional FA of 400 μg per day was shown to have enrichment in the methylation of maternally imprinted insulin-like growth factor 2 gene (IGF2) compared to those with no periconceptional maternal FA [52]. In addition, several epidemiologic and molecular evidences also link folate supplementation and epigenetic alteration by DNA methylation with neural growth and recovery, including the activation of folate receptor *(Folr1),* in spinal cord regeneration [53],[54]. In an attempt to understand the FA induced epigenetic mechanism to rescue neural tube closure, a recent study in Splotch embryos (*Sp−/−)* has also shown that maternal intake of folate prior to conception decreases the H3K27 methylation marks and remodels the chromatin on *Hes1* and *Neurog2* promoters, genes that are essential for neural tube development [55]. Recently, our study has shown that FA supplementation dysregulates expressions of several genes including *FMR1* in lymphoblastoid cells [56], and a follow up study in a mouse model has also identified widespread alteration in the methylation pattern of the brain epigenome in offspring from high maternal FA during gestation [57]. The alterations in the methylation pattern were exhibited both in CpG and non-CpG regions resulting in differences in the expression of several key developmental and imprinted genes. In addition, we also found that the methylation and expression of several genes are altered in a gender-specific manner. Thus, it is clear that folate plays a key role in epigenetic regulation of fetal developmental programming. In the future, more studies on the role of folate deficiency or over supplementation on epigenetic alterations will establish causality of the amount of FA and DNA methylation in diseases.

### Folate intake and concern about potential adverse effect

The clinical significance of the chronic or high intake of FA is not well established. Post fortification epidemiological studies have reported an increase of approximately twice the amount in the intake of FA than previously projected. Concern has been raised regarding the potential health effects, since in addition to the fortified products there is prevalence of using widespread supplementation including over-the counter prenatal vitamins as well as energy drinks which are substantially enriched with various vitamins [58],[59]. Recently, our study in the mouse model has found that ten-fold increase in maternal FA supplementation during gestation altered the expression of several genes in the frontal cortex of day old pups [60]. Moreover, continuation of such higher amounts of FA throughout the post-weaning period exhibited alterations in behaviors compared to offspring from mothers having lower doses of gestational FA supplementation. Mechanistically, such changes of behavioral outcomes may possibly result from alterations of gene expression as a result of aberrant methylation.

Intriguingly, results from several studies also suggested that folate supplementation can induce aberrant patterns of DNA methylation, and mechanistically may play a dual role in carcinogenesis. FA supplementation may prevent the early lesions, or potentially harm by enhancing the progression of established preneoplastic lesions [61]. Studies in rodent models -have shown that supplementation of FA promotes the progression of mammary tumor, and supporting this view a study in a genetically engineered mouse model of a human cancer has shown that FA deficiency during the peri-gestational period protects or decreases medulloblastoma formation [62],[63]. However, a meta-analysis of data conducted on 50,000 individuals to assess the effects of FA found that FA supplementation does not substantially increase or decrease incidence of site-specific cancer during the first 5 years of treatment (RR = 1.06, 95% CI 0.99-1.13) in comparison to placebo [64]. Moreover, a study from children participating in the Northern California Childhood Leukemia Study (NCCLS) further revealed no significant association of folate concentration at birth with childhood acute myeloid leukemia (Additional file 1: Table S1). In contrast, several RCT and meta-anlayis have reported that prenatal multivitamins containing FA -are associated with a significant protective effect on pediatric cancers: leukemia, pediatric brain tumors and neuroblastoma [65], (Additional file 1: Table S1). In addition, recent ecological studies provided support for a decrease in Wilms tumour, neuroblastoma, primitive neuroectodermal tumours and ependymomas after Canadian and United States FA fortification [66],[67], (Additional file 1: Table S1).

Although controversial, over-supplementation is also reported to be involved in certain chronic disease and found not to reduce cardiovascular disease [68],[69]. In addition, acute folate intake is also found to result in significant down-regulation of folate transporters in kidney, and thus dysregulated the renal folate uptake process [70]. Moreover, several RCT and observational studies suggested that maternal intake of multivitamins including FA during pregnancy may modulate pregnancy related outcomes [71]-[75] including developmental outcome of offspring (Additional file 1: Table S1).

The causal link between the maternal FA supplementation and the development of childhood asthma has been of interest as asthma is considered to be an interaction of both genetic and environmental risk factors, and concern has arisen as epidemiological studies have also shown that increased folate in pregnancy may influence poor respiratory health in children [76],[77]. Several studies, including RCT and observational studies were conducted to reveal such associations, however conflicting results were found in these studies. While some studies found positive association between FA exposure and increase in risk of childhood asthma, other studies found no such association (Additional file 1: Table S1). In addition, studies in humans, also reported to have found higher blood folate concentration of unmetabolized FA and naturally occurring folates [78].

To gain a better understanding if maternal supplementation of FA modulates pregnancy related outcomes, much focus has been given to reveal the role of FA supplementation in the increased incidence of dizygotic twining. This followed after the report of a Swedish study suggested a possible association of FA supplementation with the increase in the twining rate [79]. A meta-analysis using the Food Standards Australia New Zealand (FSANZ) framework by Muggli et al. [80] has suggested that the hypothesis of the increase in dizygotic twins is still to be demonstrated (OR = 1.26, 95% CI 0.91-1.73 for pre-conceptional supplementation and dizygotic twins; OR = 1.02, 95% CI 0.85-1.24 for overall twins), and, if true, it would only cause a very limited increase. However, Berry et al. reported that the association of FA with an increase in dizygotic twining as reported by the Swedish study has probably led to false findings based on the reported 40% misclassification of the use of *in vitro* fertilization [81]. This was further supported by a Norwegian study that found no evidence for an association between preconceptional folate supplements and twinning after exclusion of known *in vitro* fertilization pregnancies, and accounting for under-reporting of both *in vitro* fertilization pregnancies and folate use [82].

Thus it is clear that FA intake during pregnancy and during daily life plays a significant role in modulating gene expression and disease related outcome. Considering the important role of FA in several cellular process, including epigenetic modulation and reducing the incidence of NTDs (Additional file 1: Table S1), the dose, timing (pre-conceptional/peri-conceptional/in-pregnancy), and source of folate intervention during pregnancy and throughout the life time may be critical. In the future, more clinical and basic studies to decipher the link between over supplementation and normal development will help us to understand the discordances between benefit and possible harm.

### Maternal folate intake and health outcomes in children - a brief systemic review of recent cohorts study

For a better understanding of the effect of maternal FA, we systematically reviewed recent published literature (2011–2014) in order to assess the outcome of maternal FA supplementation on the health of newborn infants [83]-[134] (Additional file 1: Table S1). While results of several cohort and observational studies in USA, Canada, Chile, Australia, several countries in Europe and Asia have reported the clinical significance of FA supplementation, the direction of the beneficial effect was not in favorable terms in all the cases. Therefore, several countries have mandatory regulated FA fortifications, and despite its efficacy there is no universal agreement based on the published data to date [135]. The concern regarding the appropriate dose and potential side effects are still a matter of debate [16],[136]. As maternal FA can induce potential epigenetic effects on the genome of the offspring which may vary with the metabolic ability of individual race, sex, geographical locations or interactions with other nutrients, one possible reason of inconsistency between studies may be due to differences in the design of the study. In the future there is definitely a need of global collaboration to accumulate scientific evidence from a clinical perspective, and to interpret these intervention studies and potential effect in large cohorts.

## Conclusion

**Figure 2:**
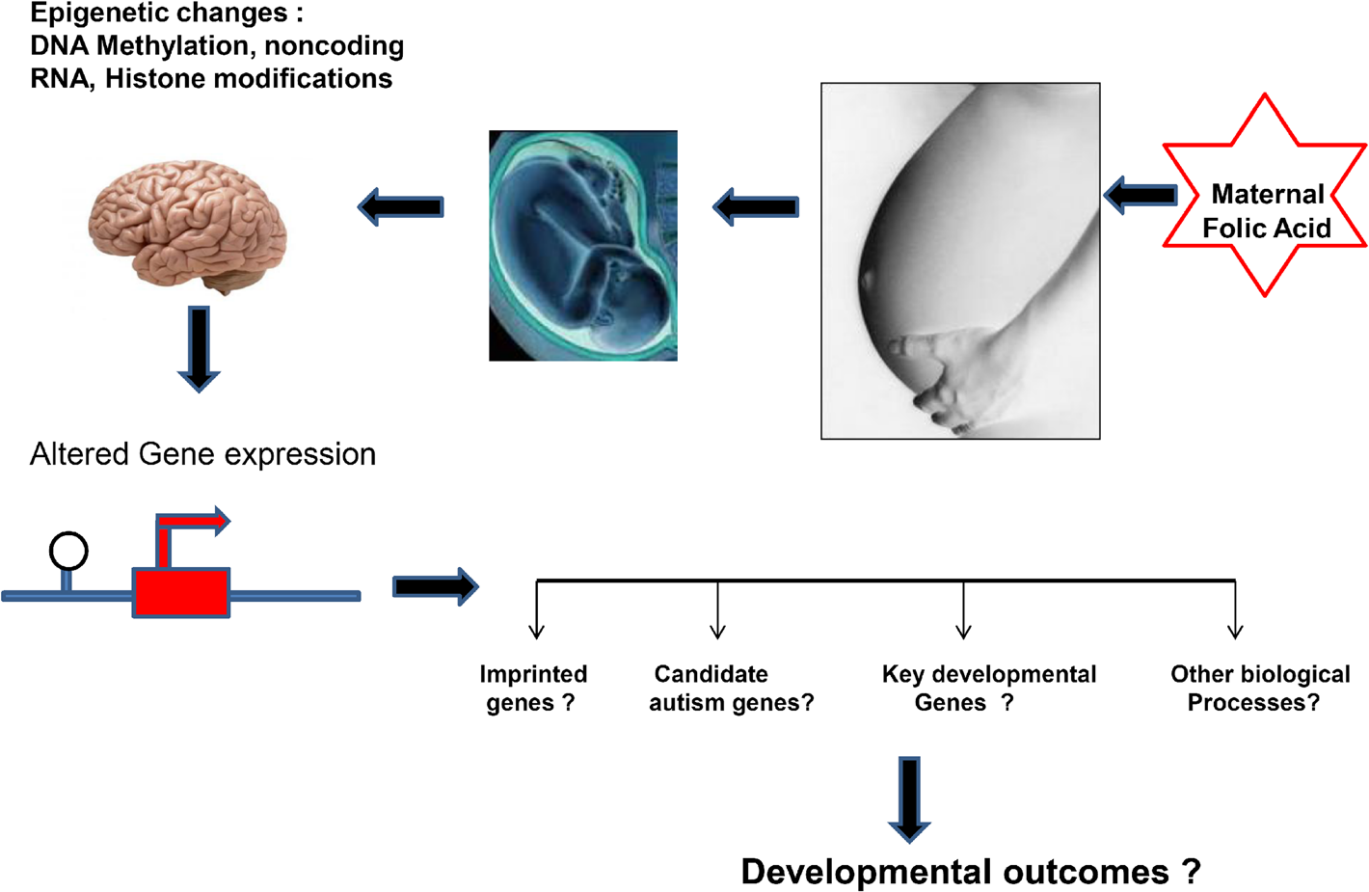
**A representative integrative model of possible epigenetic influence on pregnancy outcomes.** Maternal intake of FA may result in epigenetic modulation in the offspring brain methylome with overall or site specific alterations of methylation in genomic DNA, non-coding RNA and histone modifications. These effects may alter gene expression of several imprinted, candidate autism susceptibility and key developmental genes. Such changes may impact other biological processes, and associate with the overall developmental outcome. Scientific artwork adapted from [137],[138].

The clinical application of FA supplementation/intake to prevent NTDs has been well proven for the last 20–25 years (Additional file 1: Table S1). However considering the concern with the level of folate concentration following post-fortifications, it is of interest to explore if FA exposure in significant sections of the population is influencing other normal biological processes, such as the brain’s development (Figure 2). Determining the level and distribution of the methylation profile of the brain epigenome may reveal the mechanism and downstream consequences of various neuropsychiatric and imprinted disorders including autism. Moreover as the level of folate status can influence methylation, in the future more studies are needed to explore the systemic differences in the DNA methylation profile in relation to timing and dose between different populations and between genders. Studies and careful monitoring of the consequences of FA intake in global perspectives will help clinicians to determine a proper therapeutic strategy and the best preventive measures to improve the overall public health, moreover to precisely differentiate the evaluation of this vitamin in nutrition, in fortification and in supplementation.

## Abbreviations

FA: Folic acid

NTD: Neural tube defect

CDC: Centers for disease control and prevention

RCT: Randomized control trial

SAM: S-adenosyl- methionine

SAH: S-adenosylhomocysteine

DNMTs: DNA methyltransferase

5-methylTHF: 5-methyl tetrahydrofolate

THF: Tetrahydrofolate

MTHFR: Methylenetetrahydrofolate reductase

MTRR: Methionine synthase reductase

MTR: Methionine synthase

RFC: Reduced folate carrier

SNPs: Single-nucleotide polymorphisms

IGF2: Insulin-like growth factor 2 gene

BMI: Body mass index

TRoCA: Tabriz registry of congenital anomalies

LiST: Live saved tool

PTB: Preterm birth

SGA-W: Small-for-gestational age for weight

SGA-H: Small-for-gestational age for height

ALL: Acute lymphoblastic leukemia

AML: Acute myeloid leukemia

CBT: Childhood brain tumors

## Competing interests

The authors declare that they have no competing interests.

## Authors’ contributions

SB conceptualized the content, and wrote the manuscript; SK assisted with the revision of manuscript; MAJ conceptualized the content and critically revised the manuscript. All authors read and approved the final manuscript.

## Additional file

## Supplementary Material

Additional file 1: Table S1. Studies of maternal folate intake and health outcomes in children. Click here for file
